# Is the Hierarchy of Loss in Functional Ability Evident in Midlife? Findings from a British Birth Cohort

**DOI:** 10.1371/journal.pone.0155815

**Published:** 2016-05-17

**Authors:** Elizabeth G. Wloch, Diana Kuh, Rachel Cooper

**Affiliations:** MRC Unit for Lifelong Health and Ageing at UCL, London, United Kingdom; Cardiff University, UNITED KINGDOM

## Abstract

**Background:**

Difficulties performing a range of physical tasks of daily living have been shown to develop in older populations in a typically observed sequence, known as the hierarchy of loss. Nearly all previous research has been undertaken using populations aged over 75. This study aimed to use cross-sectional and longitudinal data to test for evidence of the hierarchy of loss from midlife onwards.

**Methods:**

The prevalence of reported difficulty undertaking 16 physical tasks in the MRC National Survey of Health and Development at age 60–64 were calculated, with Mokken scaling used to confirm the hierarchical order. Logistic regression was used to calculate the odds ratios of reporting difficulty performing tasks at the bottom of the hierarchy (i.e. feeding, washing and/or toileting) at age 60–64 by reported difficulty at the top of the hierarchy (i.e. gripping, walking and/or stair climbing) at age 43.

**Results:**

At age 60–64, tasks associated with balance, strength and co-ordination, such as climbing stairs, were the first tasks participants reported difficulty with and tasks associated with upper limb mobility, such as feeding yourself, were the last. In a fully-adjusted model, participants who reported difficulty at the top of the hierarchy at age 43 were 2.85 (95% CI: 1.45–5.60) times more likely to report difficulty with tasks at the bottom of the hierarchy at age 60–64.

**Conclusion:**

This study presents evidence of the hierarchy of loss in a younger population than previously observed suggesting that targeted interventions to prevent functional decline should not be delayed until old age.

## Introduction

Maintaining physical capability, the ability to perform the physical tasks of daily living, is considered to be an important component of healthy ageing by older people,[1,2] and researchers.[3–5] Evidence suggests that from midlife onwards age-related declines in physical capability are observed in both objective and self-reported measures.[4,6–8] When focusing on self-reported measures, difficulties performing physical tasks of daily living develop in a typically observed sequence, described as the hierarchy of loss.[9,10] Generally, the first tasks which people report difficulty with are those associated with strength, balance and co-ordination, and the last tasks are those incorporating manual dexterity.[11] This pattern has been consistently observed across populations in Europe, Asia and North America,[11–14] in both cross-sectional and longitudinal studies.[15–17] Despite sex differences in levels and rates of decline in physical capability,[6,7] the hierarchy follows a similar pattern in men and women, with only a few differences observed in the ordering of tasks involving strength.[10,18] This hierarchy of loss is thought to be independent of pathological causes,[9] although rate of progression through the hierarchy may be affected by the number of co-morbidities.[13]

Although some previous work exploring the hierarchy of loss has focused specifically on mobility tasks,[11,18] most studies have included tasks covering mobility and basic and instrumental activities of daily living to incorporate the full range of tasks representing physical capability. However, most studies have included a limited number of tasks (usually ≤10),[11,13–16,18] with the most comprehensive study including 18 tasks.[19] Further, nearly all research in this area has focused on populations over the age of 75, by which time a significant proportion of the population have already developed difficulties with multiple tasks. By exploring the hierarchy of loss at younger ages, it may be possible not only to establish the age at which the process begins, but also to identify individuals whilst they are in earlier stages of decline, at which point intervention may be more effective at preventing further decline and promoting recovery.[11,14,18]

The overall aim of this study was to examine the hierarchy of loss in physical capability in a British birth cohort, the MRC National Survey of Health and Development (NSHD), between ages 43 and 60–64 years. Strengths of this dataset include the assessment from age 43 years onwards of a range of physical tasks covering mobility, basic activities of daily living (ADLs) and instrumental activities of daily living (IADLs). It thus incorporates the full range of potentially relevant aspects of physical capability associated with maintenance of independence. Both cross-sectional and longitudinal data were used to determine if consistency could be found between these two approaches.[11] The first objective was to establish the hierarchy of loss at age 60–64 years. The second objective was to establish whether the hierarchical order observed in the cross-sectional analysis at age 60–64 would be reflected in longitudinal prevalence estimates, with tasks found to be at the top of the hierarchy at age 60–64 expected to have the greatest increase in levels of reported difficulty from age 43. The final objective was to test for evidence of progression through the hierarchy over time.

## Materials and Methods

The NSHD is a nationally representative British birth cohort, based on a socially stratified sample of 5362 singleton births that occurred during one week in March 1946 in mainland Britain. Nurse home visits were conducted at ages 43 and 53, and a clinic or home visit was conducted when participants were aged 60–64.[20,21] At this last follow-up, 2856 study participants were known to be alive and living in mainland Britain of whom 2229 completed an assessment (1690 at one of six clinical research facilities and 539 at home).[20,22] Invitations to this assessment were not sent to those who had died (n = 778), who were living abroad (n = 570), had previously withdrawn from the study (n = 594) or had been lost to follow-up (n = 564). All participants have provided written informed consent; data collections at ages 43, 53 and 60–64 years were approved by the Joint UCL/UCLH Committee on the Ethics of Human Research (43y), North Thames Multi-Centre Research Ethics Committee (53y) and the Central Manchester Local Research Ethics Committee and the Scotland A Research Ethics Committee (60-64y), respectively. Bona fide researchers can apply to access the NSHD data via a standard application procedure (further details available at: http://www.nshd.mrc.ac.uk/data.aspx).

### Physical capability

Self-reported physical capability was ascertained at ages 43, 53 and 60–64. Participants were asked to report whether they had difficulty performing a number of different tasks at each age (Table 1). For the purposes of these analyses each variable has been dichotomised: no difficulty versus any difficulty.

**Table 1:** Self-reported difficulty with physical capability tasks assessed in the MRC NSHD at ages 43, 53 and 60–64 years.

Tasks	Age (y)		
	43	53	60–64
Walk 400m	✓	✓	✓
Climb flight of 12 stairs	✓	✓	✓
Grip (holding, gripping or turning things)	✓	✓	✓
Bend down and straighten up	✓		✓
Maintain balance (need to hold onto something)	✓		✓
Shop (carry heavy load in each hand)	✓		✓
Bath and shower	✓		✓
Get in and out of a chair	✓		✓
Get in and out of bed	✓		✓
Dress and undress	✓		✓
Get to the toilet	✓		✓
Use toilet	✓		✓
Wash hands and face	✓		✓
Feed self (including cutting food)	✓		✓
Prepare hot meal			✓
Heavy housework			✓

Note: The questions used within NSHD are based on the Office of Population Censuses and Surveys (Martin J, Meltzer H, Elliot D. OPCS Surveys of Disability in Great Britain, Report 1: The Prevalence of disability among adults. London: HMSO; 1988)

### Covariates

Potential confounders were identified *a priori*. Three socio-demographic factors were selected: sex, education and occupation. Highest educational level attained by age 26 was categorised into five groups: degree or higher; A levels or their equivalent (usually attained at age 18); O levels or their equivalent (usually attained at age 16); CSE, clerical course or equivalent and; none. Occupation was recorded at age 53 (or the most recent measure available in adulthood (n = 83)) and categorised using the Registrar General’s social classification into three groups: high (I or II); medium (III manual or non-manual) and; low (IV or V).

Two lifestyle factors were selected: smoking history and participation in leisure time physical activity (LTPA). Smoking status, recorded throughout adulthood was categorised as current, former or never-smoker at age 60–64. Study members were asked how frequently they had participated in any sports, vigorous leisure activities or exercise in their spare time, per month at age 43 or in the past four weeks at ages 53 and 60–64. As in previous analyses,[23] at each age individuals were categorised as inactive (reported no participation), moderately active (participated 1–4 times) or most active (participated ≥5 times) and scored 0–2 respectively. These values were then summed to produce a cumulative score of participation in LTPA (ranging from 0 (inactive at all 3 ages) to 6 (most active at all 3 ages)).

Three indicators of health status were selected: obesity, depression and respiratory disease. For each of these variables three response categories were identified that took account of baseline and incident health status: never experienced health condition; present at age 43 and; present by age 60–64. Body mass index was calculated at ages 43 and 60–64 using measured height and weight, then dichotomised using a standard cutpoint for obesity (≥30 kg/m^2^). Symptoms of anxiety and depression were identified using the Psychiatry Symptom Frequency scale at age 43[24] and the General Health Questionnaire-28 at age 60–64.[25] For both scales “caseness” thresholds (≥23 and ≥5, respectively), were used. The UK Medical Research Council’s standardised questions[26] were used to identify those with severe respiratory symptoms at ages 53 and 60–64, reflecting their experience over the past three years. Participants were considered to have severe respiratory symptoms if they reported one or more of the following: wheezy or whistling chest most days or nights, usually bringing up phlegm or coughing in the morning or during the day or night in winter for at least three months each year, or a chest infection that kept them off work or indoors for more than a week.

### Statistical analysis

To address the first study objective, to establish the hierarchy of loss at age 60–64, descriptive analyses were used to examine the prevalence of reported difficulty performing each of the 16 physical capability tasks at this age. These prevalence estimates were plotted on figures ordered from highest to lowest prevalence. For each task, the median number of other tasks that participants reported difficulty with if they had reported difficulty with that specified task was calculated and plotted on the same figure. These descriptive analyses were stratified by sex to account for the sex differences in levels of physical capability[6] and restricted to those with complete data (N = 2063). Sex-specific Mokken scales were used to confirm the hierarchical order of the tasks at age 60–64, with assumptions of unidimensionality, single and double monotonicity tested.[10,17] The Mokken scales produced take a probabilistic approach with the hierarchical order within the scale considered to be strong if the Loevinger Scalability Coefficient (H) >0.5.

The second study objective, to establish whether the hierarchical order observed in the cross-sectional analysis would be reflected in longitudinal prevalence estimates, was addressed by calculating the sex-specific prevalence of reported difficulty for the three tasks available at ages 43, 53 and 60–64 (gripping, walking and climbing stairs). This analysis was restricted to those who responded at all three ages (N = 2046).

To address the final objective, to test for evidence of progression through the hierarchy, logistic regression was then used to calculate the odds ratio of reporting difficulty performing tasks at the bottom of the hierarchy (i.e. feeding, washing and/or toileting) at age 60–64 by reported difficulty performing tasks at the top of the hierarchy (i.e. gripping, walking and/or stair climbing) at age 43 (N = 2106). Participants who reported difficulty with tasks at the bottom of the hierarchy at age 43 were excluded (N = 6). An initial sex-adjusted model was run, with sex interaction formally tested. Each group of covariates were then added to the model separately before all covariates were incorporated into a fully-adjusted model. This was then repeated stratified by sex. In order to minimise the potential risk of bias introduced by missing data, and to maintain statistical power, multiple imputation chained equations were used to impute missing values for covariates (educational level (n = 99), occupation (n = 1), smoking (n = 20), LTPA (n = 139), obesity (n = 33), depression (n = 57) and respiratory disease (n = 204)). The logistic regression analyses were run across 15 imputed datasets and combined using Rubin’s rules.[27]

All analyses were conducted using Stata version 12.

## Results

**Fig 1:**
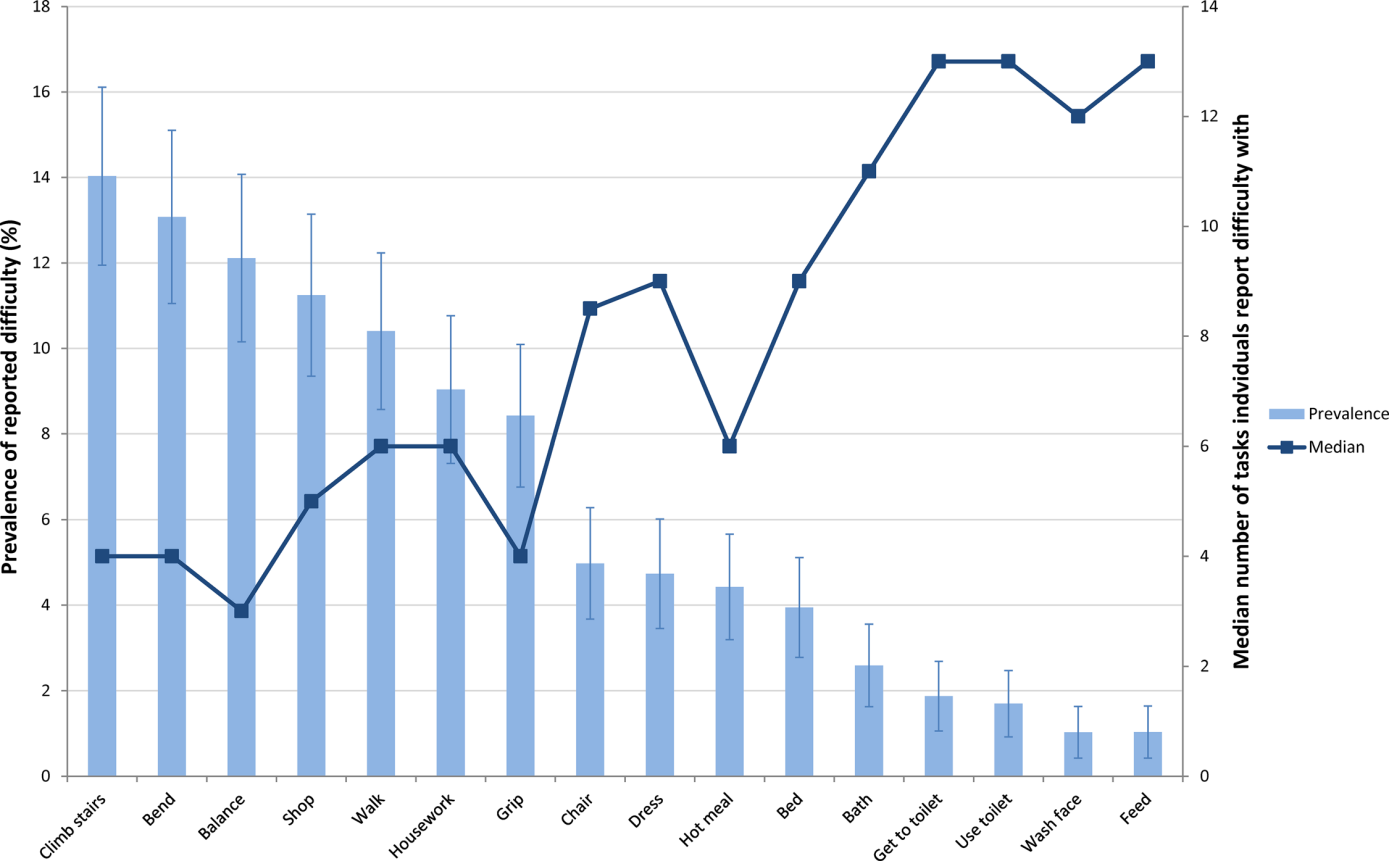
Prevalence of reported difficulty with 16 physical capability tasks at age 60–64 among men in the MRC NSHD (N = 1001). For further details of tasks assessed see Table 1.

**Fig 2:**
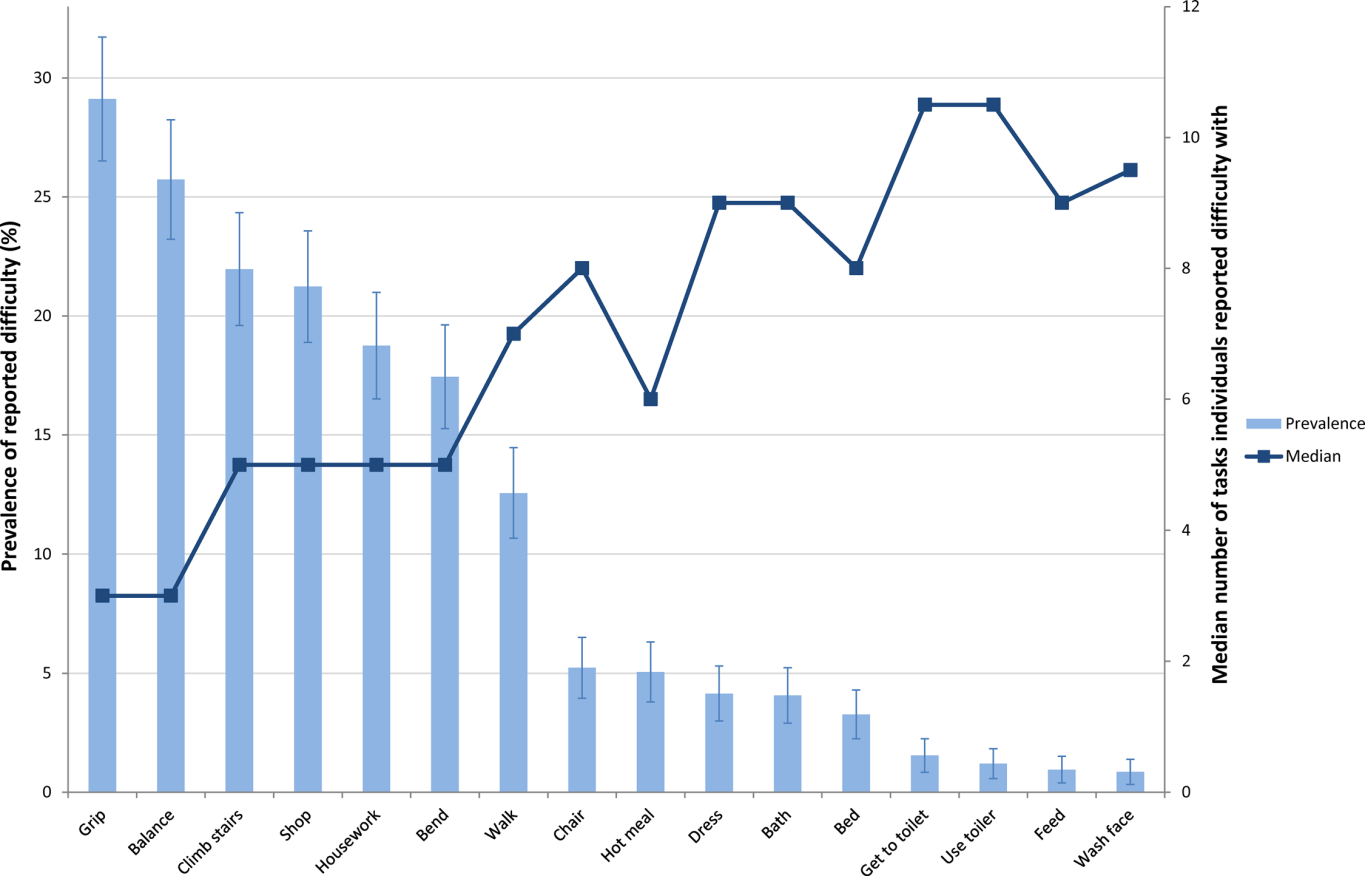
Prevalence of reported difficulty with 16 physical capability tasks at age 60–64 among women in the MRC NSHD (N = 1062). For further details of tasks assessed see Table 1.

The characteristics of the NSHD participants included in analyses are shown in Table 2. In this community dwelling sample at age 60–64, 30% of men and 55% of women reported difficulty with at least one of the 16 physical capability tasks. Tasks associated with balance, strength and co-ordination, such as climbing stairs and carrying heavy shopping, were found to have the highest prevalence of reported difficulty for both men and women (Figs 1 and 2). For these tasks, more women reported difficulty than men, with over 20% of women and 10% of men reporting difficulty with each of the four most prevalent tasks. Tasks associated with upper limb mobility, such as feeding and washing hands and face, had the lowest reported prevalence of difficulty, with less than 1% of men and women reporting difficulty with these tasks. Among both men and women a general trend was observed whereby the lower the prevalence of reported difficulty for a specific task the greater the median number of other tasks that individuals reported difficulty with.

**Table 2:** Characteristics of participants from the MRC National Survey of Health and Development (based on sample included in logistic regression analyses, maximum N = 2106).

	N* (%)	
	Men	Women
**Maximum N**	1000 (47.5)	1106 (52.5)
***Indicators of socioeconomic position***		
**Highest educational level attained by age 26**		
Degree or higher	165 (17.4)	66 (6.3)
A levels or equivalent	285 (30.0)	282 (26.7)
O levels or equivalent	144 (15.1)	284 (26.9)
CSE, clerical course or equivalent	49 (5.2)	97 (9.2)
None	308 (32.4)	327 (31.0)
**Occupational class at age 53**		
High (I or II)	559 (55.9)	422 (38.2)
Medium (III)	347 (34.7)	481 (43.5)
Low (IV or V)	94 (9.40)	202 (18.3)
***Health behaviours***		
**Smoking status at age 60–64**		
Never smoker	277 (28.0)	387 (35.3)
Ex-smoker	592 (59.9)	580 (52.8)
Current smoker	119 (12.0)	131 (11.9)
**Cumulative leisure time physical activity score**^**†**^ **(mean (SD))**	2.36 (1.92)	2.21 (1.88)
***Health status between ages 43 and 60–64***		
**Obesity**		
Not obese at either age	699 (70.8)	737 (67.9)
Became obese between ages 43 and 60–64	198 (20.0)	222 (20.4)
Obese at age 43	90 (9.1)	127 (11.7)
**Symptoms of anxiety and depression**		
No symptoms at either age	806 (82.7)	757 (70.5)
Developed symptoms between ages 43 and 60–64	86 (8.8)	138 (12.9)
Symptoms present at age 43	83 (8.5)	179 (16.7)
**Severe respiratory symptoms**		
No symptoms at either age	661 (73.4)	738 (73.7)
Developed symptoms between ages 43 and 60–64	91 (10.1)	87 (8.7)
Symptoms present at age 43	148 (16.4)	177 (17.7)

* Total N varies due to missing data on covariates

† Cumulative leisure time physical activity score derived by assigning those classified as inactive a value of 0, those as moderately active a value of 1, and those as most active a value of 2 at ages 43, 53 and 60–64 and then summing the values for the three ages, range 0 (inactive at all 3 ages) to 6 (most active at all 3 ages). For this score N = 922 men, 1045 women

The two sex-specific Mokken scales confirmed the hierarchical order observed, with scales in both sexes producing Loevinger’s scalability coefficients >0.5 (H = 0.54 (women), H = 0.59 (men)).

**Fig 3:**
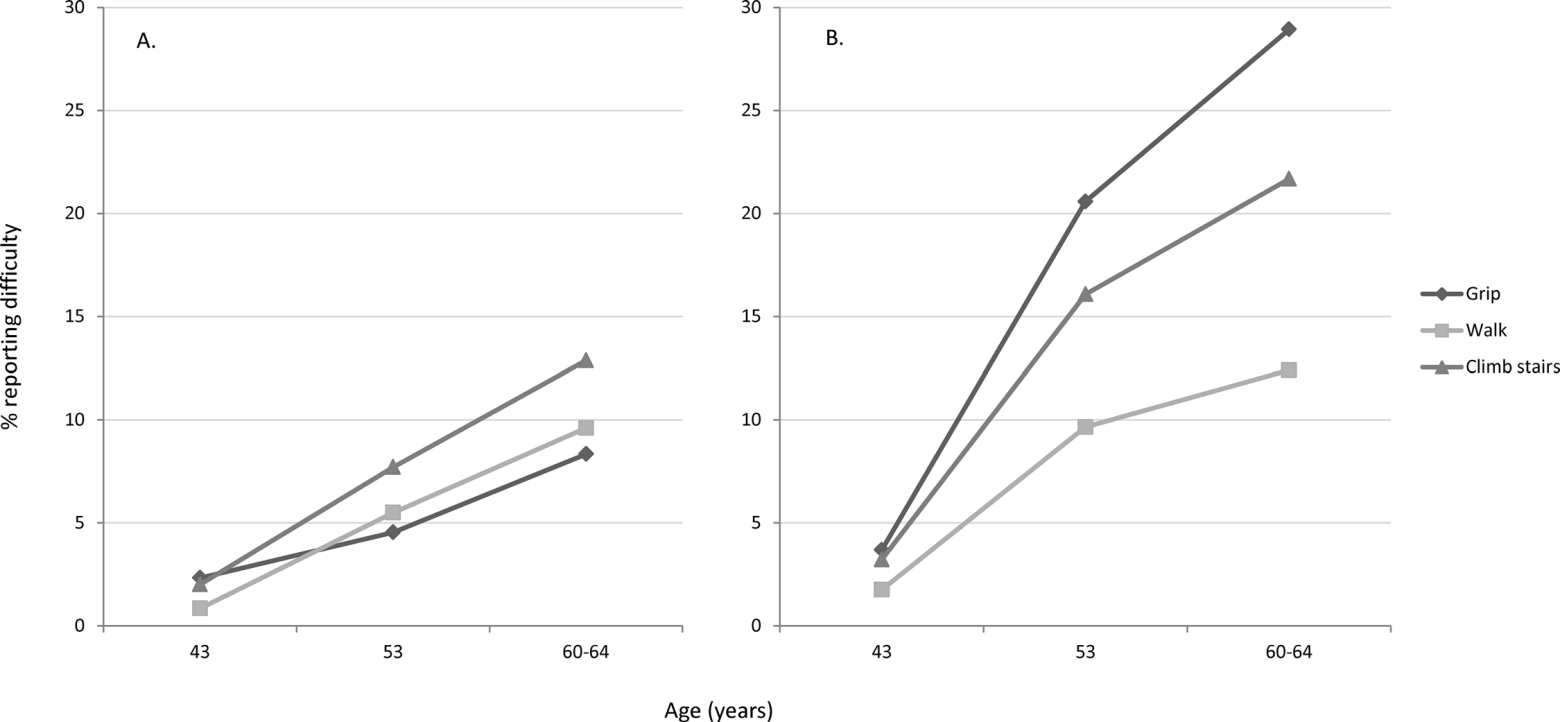
Prevalence of reported difficulty gripping, walking 400m and climbing stairs at ages 43, 53 and 60–64 ((A) Men (N = 957) (B) Women (N = 1089)).

From age 43 onwards a diverging trend in the prevalence of reported difficulty was observed for the three tasks assessed at all three ages (Fig 3). For women, the prevalence of difficulty performing the three tasks increased from a range of 2–4% at age 43 to a range of 12–29% by age 60–64, with the greatest increase in prevalence found for difficulty gripping and the smallest increase in difficulty walking. The trend was less marked in men.

In a sex-adjusted model, those individuals who reported difficulty with at least one task at the top of the hierarchy (i.e. gripping, walking and/or climbing stairs) at age 43, had 4.96 (95% CI: 2.70–9.11) times higher odds of reporting difficulty with at least one task at the bottom of the hierarchy (i.e. feeding, washing and/or toileting) at age 60–64, when compared with individuals who reported no difficulty at age 43 (Table 3). Adjustment for each group of covariates attenuated the relationship; fully-adjusted OR = 2.85 (95% CI: 1.45–5.60). When formally tested there was no evidence of sex interaction (p = 0.14). However, when analyses were repeated stratified by sex the association was much stronger in women (Table 3), with a fully-adjusted OR = 5.28 (95% CI: 2.19–12.73) compared with 1.54 (95% CI: 0.45–5.29) for men.

**Table 3:** Odds ratios of progressing through the hierarchy of loss between ages 43 and 60–64 years in the MRC NSHD.

	Reported difficulty at the top of the hierarchy at age 43*	Model^†^	Odds Ratio (95% CI) of reported difficulty at the bottom of hierarchy at age 60–64^‡^
Men and Women	No difficulty (ref.)		1
(N = 2106)	Difficulty	1	4.96 (2.70–9.11)
		2	4.33 (2.34–8.02)
		3	3.16 (1.63–6.14)
		4	4.04 (2.16–7.56)
		5	2.85 (1.45–5.60)
Men (N = 1000)	No difficulty (ref.)		1
	Difficulty	1	2.72 (0.91–8.09)
		2	2.29 (0.76–6.91)
		3	1.60 (0.50–5.18)
		4	2.50 (0.82–7.62)
		5	1.54 (0.45–5.29)
Women (N = 1106)	No difficulty (ref.)		1
	Difficulty	1	7.17 (3.36–15.34)
		2	6.51 (3.01–14.07)
		3	5.58 (2.35–13.26)
		4	5.49 (2.51–12.00)
		5	5.28 (2.19–12.73)

*117 men and 145 women reported difficulty gripping, walking and/or stair climbing at age 43

^†^ Model 1: Sex-adjusted (if sexes are combined) or unadjusted (if analyses sex-specific)

Model 2: Model 1 plus socioeconomic factors (education and occupational class)

Model 3: Model 1 plus incident health variables (depression, obesity and respiratory disease)

Model 4: Model 1 plus health behaviours (smoking and leisure time physical activity)

Model 5: Fully-adjusted model

^‡^ OR are combined estimates from models run across 15 imputed datasets

31 men and 34 women reported difficulty feeding, washing and/or toileting at age 60–64

## Discussion

This study presents findings from a relatively large nationally representative British sample, to provide evidence that the hierarchy of loss in physical capability exists at younger ages than previously reported. This evidence has been demonstrated using both cross-sectional and longitudinal approaches. The descriptive analysis of data from age 60–64, suggests that individuals first report difficulty with tasks associated with strength, balance and coordination, before progressing through the hierarchy to reported difficulty with tasks requiring manual dexterity. This hierarchical ordering of tasks was confirmed cross-sectionally using Mokken scaling and, longitudinally, with the greatest increases in prevalence of reported difficulty between ages 43, 53 and 60–64 years observed for the tasks at the top of the hierarchy (i.e. climbing stairs (men) and gripping (women)). Further evidence that the hierarchy of loss exists at younger ages was found when exploring the progression of individuals through the hierarchy. If individuals reported difficulty with tasks at the top of the hierarchy at age 43 they were nearly three times more likely to report difficulties at the bottom of the hierarchy by age 60–64, compared to those with no difficulty at age 43. This association was particularly strong among women.

One strength of this study is its inclusion of more tasks than most previous work which facilitates comparisons with other studies. When shopping, walking 400m and stair climbing were included in studies they were consistently identified within the literature as among the first tasks with which individuals reported difficulty.[10,14,17] The results of this study support this observation but also demonstrate the importance of additional tasks towards the top of the hierarchy, such as balancing and bending. In line with previous findings, including some other British studies,[10,16] difficulty washing and feeding were identified as the last tasks with which individuals reported difficulty.[9,10,12,15,16] It is interesting to note that this hierarchical order is consistent even at younger ages when prevalence of difficulty undertaking some tasks is very low.

As expected, prevalence of difficulty with any specific task was higher among women than men and, women were more likely than men to report difficulty with a greater number of tasks. This may explain why the association between difficulty with tasks at the top of the hierarchy at age 43 and progression of loss by age 60–64 was much stronger for women than men. However, despite this, the hierarchical order of tasks presented was generally similar in both sexes, corresponding with previous findings.[10,15,16,18] The slight differences observed were in tasks associated with strength, most noticeably gripping, and this may reflect known biological differences between men and women.[28]

The reason for finding a hierarchical ordering of tasks can be partly explained by age-related changes in underlying physiological systems. We consider the tasks towards the start of the hierarchy to generally be more complex and to depend on the interaction of multiple physiological systems. For example walking 400m relies on the normal functioning of multiple systems including the nervous and cardio-respiratory systems, in addition to the musculoskeletal system.[29] Such tasks are susceptible to changes in any one of the systems involved. Consequently these more complex tasks are often those which individuals first report difficulty with.

The findings of this study are applicable at the population level and inferences should be made at the individual level with caution. The majority of individuals are believed to have followed the hierarchical order presented, as demonstrated by strong Mokken scales and diverging longitudinal trends. However, not all individuals who reported difficulty at age 43 will have progressed through the hierarchy by age 60–64, due to the dynamic nature of physical capability decline.[11,14] Physical capability decline is not inevitable and may be temporary or reversible, particularly at the start of the transition. Although recovery rates are reasonably low, there is some evidence to suggest that rates of recovery may be higher for those tasks with which individuals first report difficulty.[16]

There are several methodological considerations when interpreting this study’s findings. Self-reported measures of physical capability were chosen over objective performance-based counterparts, as they specifically capture loss of capability. While it is important to consider that an individual’s perception of ability may differ from their actual ability, one study has found the same hierarchical order whether self-reported or performance measures of physical capability were used.[14] This suggests that the influence on findings of subjectivity when using self-reported measures is limited. In addition, self-reported measures have the benefit of capturing the experience of individuals within their everyday environments, rather than their performance under standard conditions, which is relevant when considering how this research relates to policy focused on the maintenance of independence. A key strength of these analyses is the availability of repeat data on functional limitations from age 43 years. This enabled the longitudinal study of loss of functional ability during an earlier phase of adulthood than most previous studies have investigated. However, as ages at onset of limitations were not recorded and there was a 10 year interval between assessments we were unable to explore shorter-term changes in functional ability or time to event.

Tasks towards the top of the hierarchy have been highlighted as important predictors of future physical capability decline, but the challenge remains to identify a specific group of tasks for which reported difficulty can be used to formulate practical policy suggestions. Therefore, a decision was made *a priori* to base the hierarchy of loss in this paper on single items rather than the groups of tasks or domains suggested in the literature.[9,10] The aim was to add to the existing evidence base and clarify the order in which individuals report difficulty with tasks.

As in any longitudinal study there have been losses to follow up due to death, emigration and permanent refusal in the NSHD. However, the sample remained broadly representative of the general population, who were born in Britain, at age 60–64.[22] Since participants needed to have data recorded at age 60–64 to be included in analyses, it is possible that bias due to a healthy survivor effect may have been introduced. A sensitivity analysis concluded that there was little evidence of this effect with similar prevalence estimates of reported difficulty produced across all ages when incomplete data were incorporated. In addition, the influence of bias due to missing data on covariates was minimised through use of multiple imputation.

The evidence presented in this study suggests that the hierarchy of loss operates from at least the fifth decade of life. A small group of individuals have already progressed through the hierarchy to severe limitations by early old age, and this is likely to reduce their quality of life. These individuals will often require care,[15] placing a burden on families and health and social care systems. It may be possible to use the hierarchy of loss to help prioritise resources and service allocation to assist with the maintenance of independence. If attention is paid to individuals when they first report difficulty with tasks towards the top of the hierarchy it may be possible to identify those at high risk of subsequent functional decline, and consequently loss of independence. These individuals are likely to be suitable candidates for targeted intervention to prevent further decline and promote recovery.

The results of this study suggest that the first seven items in the hierarchy (gripping, balancing, stair climbing, housework, shopping, bending and walking 400m) may be suitable tasks to use when identifying high risk individuals. However, further follow-up is required to confirm that these tasks predict future functional decline in the NSHD. In addition, replication in other studies at similar ages, using standardised measures of physical capability is needed to help strengthen the evidence base. Future work may also benefit from the extension of the hierarchy of loss beyond reported difficulty to incorporate modification of tasks,[30] as this may capture functional decline at an even earlier stage, providing further opportunities for early intervention.

The findings from this British birth cohort suggest that the hierarchy of loss in functional ability is evident at younger ages than previously observed. This suggests that targeted interventions for physical capability decline should not be delayed until old age; high risk individuals may be identified in midlife at a point when the reversal or prevention of further decline may be possible. By early old age some individuals have already experienced substantial decline in functional ability and as the population ages this proportion is likely to increase unless preventative action is taken.

## Supporting Information

S1 File STROBE Statement. (DOCX)
